# *Brahmaculus* gen. nov. (Leotiomycetes, Chlorociboriaceae)

**DOI:** 10.3897/mycokeys.80.64435

**Published:** 2021-05-07

**Authors:** Peter R. Johnston, Duckchul Park, Matthew E. Smith, Alija B. Mujic, Tom W. May

**Affiliations:** 1 Manaaki Whenua–Landcare Research, Private Bag 92170, Auckland 1142, (Aotearoa) New Zealand Manaaki Whenua–Landcare Research Auckland New Zealand; 2 University of Florida, Department of Plant Pathology, Gainesville FL 32611, USA University of Florida Gainesville United States of America; 3 Royal Botanic Gardens Victoria, Melbourne, Victoria 3004, Australia Royal Botanic Gardens Victoria Melbourne Australia

**Keywords:** *
Chlorociboria
*, Cyttariaceae, fungi, molecular phylogeny, systematics, 9 new taxa

## Abstract

A second genus in Chlorociboriaceae is described here as *Brahmaculus***gen. nov.** Macroscopically distinctive, all species have bright yellow apothecia with several apothecial cups held on short branches at the tip of a long stipe. The genus is widely distributed across the Southern Hemisphere; the four new species described here include two from Chile (*B. magellanicus***sp. nov.**, *B. osornoensis***sp. nov.**) and one each from New Zealand (*B. moonlighticus***sp. nov.**) and Australia (*B. packhamiae***sp. nov.**). They differ from species referred to *Chlorociboria*, the only other genus in Chlorociboriaceae, in their terrestrial habitat and ascomata that are noticeably more hairy than the known *Chlorociboria* species, most of which have apothecia with short, macroscopically indistinct hair-like elements. Based on our analyses, *Chlorociboria* as accepted here is paraphyletic. Additional study is needed to clarify where alternative, monophyletic generic limits should be drawn and how these genera may be recognised morphologically. Also described here are three new *Chlorociboria* spp. from New Zealand (*C. metrosideri***sp. nov.**, *C. solandri***sp. nov.**, *C. subtilis***sp. nov.**), distinctive in developing on dead leaves rather than wood and in two of them not forming the green pigmentation characteristic of most *Chlorociboria* species. New Zealand specimens previously incorrectly identified as *Chlorociboria argentinensis* are provided with a new name, *C. novae-zelandiae***sp. nov.**

## Introduction

The modern-day distribution of Nothofagaceae forests of the Southern Hemisphere and their associated fungi are often explained in terms of vicariance in relation to the breakup of Gondwana (e.g. Horak 1983). This explanation has been challenged in recent years (May 2017), with their distribution now thought to be due to a complex mix of ancient vicariant and geologically more recent long distance dispersal events, with evidence from Nothofagaceae phylogeny (e.g. Knapp et al. 2005) along with the phylogeny of some of their specialised fungal associates (e.g. Peterson et al. 2010b). The importance of these forests to the vegetation of southern South America and New Zealand has meant they have been amongst the most intensively studied mycologically in these regions (McKenzie et al. 2000; Johnston et al. 2012, Gamundí et al. 2017; Romano et al. 2017a). Despite this, much of the fungal diversity in these forests remains undiscovered (e.g. Johnston et al. 2012; Romano et al. 2017b).

An example of this undiscovered diversity comes from recent collections of a beautiful, small terrestrial fungus from Nothofagaceae forests in South America, New Zealand and Australia that could not be matched to any known genus. Microscopically they had a clear affinity to Leotiomycetes. The unique apothecia are morphologically complex with a branched stipe and each branch ending in one or more cups, the hymenial surface in these cups forming a complex pattern comprising separate regions with asci and paraphyses, and with hair-like elements. Preliminary sequencing of ribosomal genes of both Australasian and South American specimens showed that these fungi are phylogenetically closely related and that they are also related to the Leotiomycetes genus *Chlorociboria*.

Here we describe four species in the newly erected genus *Brahmaculus* based on a combination of unique morphological and molecular characters. We incorporate *Brahmaculus* DNA sequences into a broad multigene Leotiomycetes phylogeny to show that these fungi represent a second genus in Chlorociboriaceae. Including *Brahmaculus* in the phylogeny makes *Chlorociboria* paraphyletic but the morphological and ecological differences between *Chlorociboria* and *Brahmaculus* species means that it is not sensible to treat them as a single genus. More intensive genetic sampling of additional *Chlorociboria* species will be needed to better resolve phylogenetic relationships within Chlorociboriaceae and to clearly define the phylogenetic and morphological limits of the genus *Chlorociboria*.

It is surprising that specimens of the morphologically spectacular *Brahmaculus* have not been collected more often in the Nothofagaceae forests of the Southern Hemisphere. Although clearly widespread geographically, these fungi presumably fruit rarely.

## Methods

### Samples

Specimens were collected during surveys of fungal diversity in Southern Hemisphere forests. Brief notes on macroscopic appearance were prepared and then the specimens dried and stored in the New Zealand Fungarium (**PDD**), National Herbarium of Victoria (**MEL**), Museo Nacional de Historia Natural (**SGO**) and the Florida Museum of Natural History (**FLAS**).

### Morphology and culturing

Microscopic examinations were made from dried material routinely rehydrated and in 3% KOH and mounted in Melzer’s Reagent, or where indicated, rehydrated and mounted in water. Vertical sections about 10 µm thick were prepared from apothecia rehydrated in 3% KOH using a freezing microtome and mounted in lactic acid. Where available, living cultures were grown from germinated ascospores and are stored in the ICMP culture collection, Manaaki Whenua–Landcare Research, Auckland.

### DNA extraction and PCR amplification

DNA was extracted from apothecia that had been placed in buffer when fresh, from dried apothecia, or from mycelium from living cultures, using a QIAamp DNA mini kit (QIAGEN, US) on the QIAcube nucleic acid extraction robot (QIAGEN, US). Amplification primers used for each of the genes were: SSU – NS1 and NS4 (White et al. 1990); ITS – ITS-1F and ITS4 (White et al. 1990; Gardes and Bruns 1993); LSU – LROR and LR5 (Bunyard et al. 1994; Vilgalys and Hester 1990); MCM7 – mcm7-709for and mcm7-1348rev (Schmitt et al. 2009); RPB1 – RPB1-Af and RPB1-Cr (Stiller and Hall 1997; Matheny et al. 2002); and RPB2 – RPB2-5f2 and fRPB2-7cR (Liu et al. 1999; Sung et al. 2007).

### Phylogenetic analyses

Two phylogenetic analyses were carried out. In the first, LSU, ITS, MCM7, RPB1 and RPB2 sequences from *Brahmaculus* specimens from South America and New Zealand, together with a set of *Chlorociboria* and *Cyttaria* specimens with multi-gene data available (Table 1), were incorporated into the alignments from Johnston et al. (2019 – data available from https://doi.org/10.7931/T5YV-BE95). *Cyttaria* was added because the analysis presented by Peterson and Pfister (2010a) suggested a relationship to Chlorociboriaceae and additional genes had recently become available for *Cyttaria nigra*. The expanded dataset was reanalysed using the same methods as Johnston et al. (2019). Briefly, genes were aligned using MAFFT (Katoh and Standley 2013), a maximum likelihood (ML) analysis of the concatenated alignments was run using IQ-TREE (Nguyen et al. 2015; Chernomor et al. 2016), using models selected by ModelFinder (Kalyaanamoorthy et al. 2017) for each partitioned gene, and ultrafast bootstrap (BS) analysis with 1000 replicates estimated branch support in the ML tree (Hoang et al. 2018). *Xylaria hypoxylon* and *Neurospora crassa* were used as outgroups.

**Table 1.** GenBank accession numbers for DNA sequences of *Brahmaculus*, *Chlorociboria* and *Cyttaria* specimens used for phylogeny in Fig. 1, and for newly generated sequences used in phylogeny in Fig. 2. Sequences generated as part of this project in bold. Data for other taxa included in the Fig. 1 phylogeny from Johnston et al. (2019), see https://doi.org/10.7931/T5YV-BE95.

Species	Voucher (T = type specimen)	SSU	ITS	LSU	MCM7	RPB1	RPB2	TEF	mtSSU	β–tubulin
*Brahmaculus magellanicus*	PDD 116650 (T)	**MW364563**	**MW364557**	**MW364560**	**MW350087**	–	**MW350085**	–	–	–
*B. moonlighticus*	PDD 112225 (T)	**MK248054**	**MK248036**	**MK248011**	**MK241483**	**MK241482**	**MK241484**	–	–	–
*B. osornoensis*	FLAS-F-65492 (T)	–	**MW575608**	–	–	–	–	–		–
*B. packhamiae*	PDD 117311 (T)	–	**MW364556**	–	–	–	–	–	–	–
*Chlorociboria aeuruginascens*	TNS-F-36241	LC434588	LC425045	LC429376	–	LC431689	LC431723	–	–	–
*C. aeuruginascens*	DSM 107184 (isolate IHIA39, genome)	PRJNA382475	PRJNA382475	PRJNA382475	PRJNA382475	PRJNA382475	PRJNA382475	PRJNA382475	PRJNA382475	PRJNA382475
C. aeuruginascens ssp. australis	ICMP 15642 (T)	JN939873	NR_119520	JN939932	JN993274	JN985222	JN985532	–	–	–
*C. aeruginosa*	AFTOL-ID 151	AY544713	DQ491501	AY544669	–	DQ471125	DQ470886	–	–	–
*C. aeruginosa*	TNS-F-13596	LC434578	LC425047	LC429383	–	LC431687	–	–	–	–
*C. aeruginella*	TAAM 198514	KX090875	MH752067	–	–	KX090769	KX090722	–	–	–
*C. argentinensis*	ICMP 16995	JN939876	EF520123	JN939930	JN993275	JN985197	JN985515	–	–	–
*C. novae-zelandiae*	ICMP 18766 (T)	JN939875	JN943456	JN939940	JN993286	JN985223	JN985514	–	–	–
*C. awakinoana*	ICMP 15631	JN939870	JN943461	JN939921	JN993273	JN985219	JN985504	–	–	–
*C. clavula*	ICMP 15634	JN939866	JN943465	JN939924	JN993299	JN985215	JN985519	–	–	–
*C. duriligna*	ICMP 18763 (T)	JN939863	JN943468	JN939934	JN993279	JN985212	JN985500	–	–	–
*C. glauca*	TAAM 198458	KX090872	–	KX090821	–	KX090766	–	–	–	–
*C. halonata*	ICMP 18764	JN939860	JN943471	JN939935	JN993296	JN985209	JN985502	–	–	–
*C. metrosideri*	ICMP 23410 (T)	–	**MW364558**	–	–	–	–	–	–	–
*C. poutoensis*	ICMP 15618	–	AY755352	–	MH700576	MH682247	–	–	–	–
*C. solandri*	ICMP 23686 (T)	–	**MW364559**	–	–	–	–	–	–	–
*C. spathulata*	ICMP 18760	JN939868	JN943463	JN939923	JN993272	JN985217	JN985530	–	–	–
*C. subtilis*	PDD 112247	–	**MH921854**	–	–	–	–	–	–	–
*Cyttaria darwinii*	FH (Peterson and Pfister 2010, isolate 40, 45)	EU107180	–	EU107209	–	–	–	EU107250	EU107236	–
*Cyttaria hariotii*	FH (Peterson and Pfister 2010, isolate 44)	EU107194	–	EU107217	–	–	–	EU107251	EU107245	–
*Cyttaria nigra*	PDD 117571	MW364564	–	MW364561	MW350086	MW363493	MW350084	MW350088	MW364562	MW350089

The second analysis used ITS sequences only, treating all four *Brahmaculus* species, together with all *Chlorociboria* species with ITS sequences available, using Cenangiaceae as the outgroup. The methods were the same as those used for the multi-gene analysis, except with the TIM2+F+I+G4 model, selected using ModelFinder.

Alignments and partitions for each of the analyses are provided through the Landcare Research – Manaaki Whenua Datastore, https://doi.org/10.7931/2xet-fc88.

## Results

### Phylogenetic analyses

**Figure 1.**
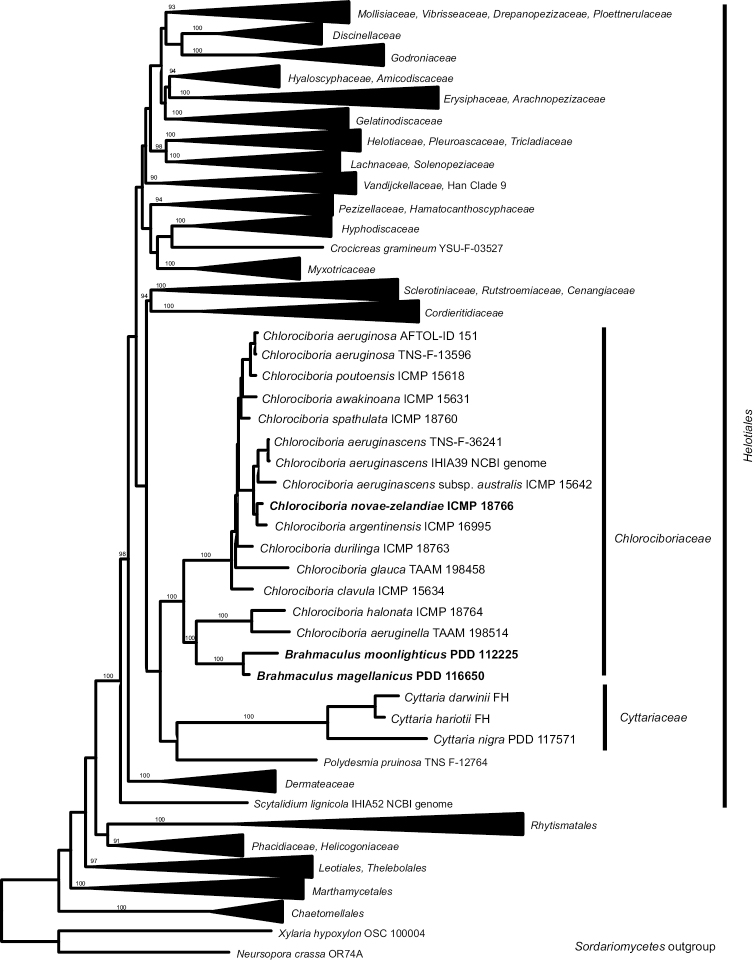
ML tree based on a multi-gene alignment, placing *Brahmaculus* within Chlorociboriaceae and both Chlorociboriaceae and Cyttariaceae in Helotiales. Taxa newly named in this paper in bold. Bootstrap values where >90%. See Methods and Table 1.

Helotiales form a strongly supported monophyletic clade, and most families accepted within this order also form strongly supported clades (Fig. 1). The family-level clades of those families clustered in collapsed clades in Fig. 1 have 100% bootstrap support. Chlorociboriaceae and Cyttariaceae are strongly supported as monophyletic but their relationship to each other, and to other basal family-level clades within the Helotiales, is poorly resolved. A fully expanded version of Fig. 1 is available as a nexus file from the Landcare Research – Manaaki Whenua Datastore, https://doi.org/10.7931/2xet-fc88.

**Figure 2.**
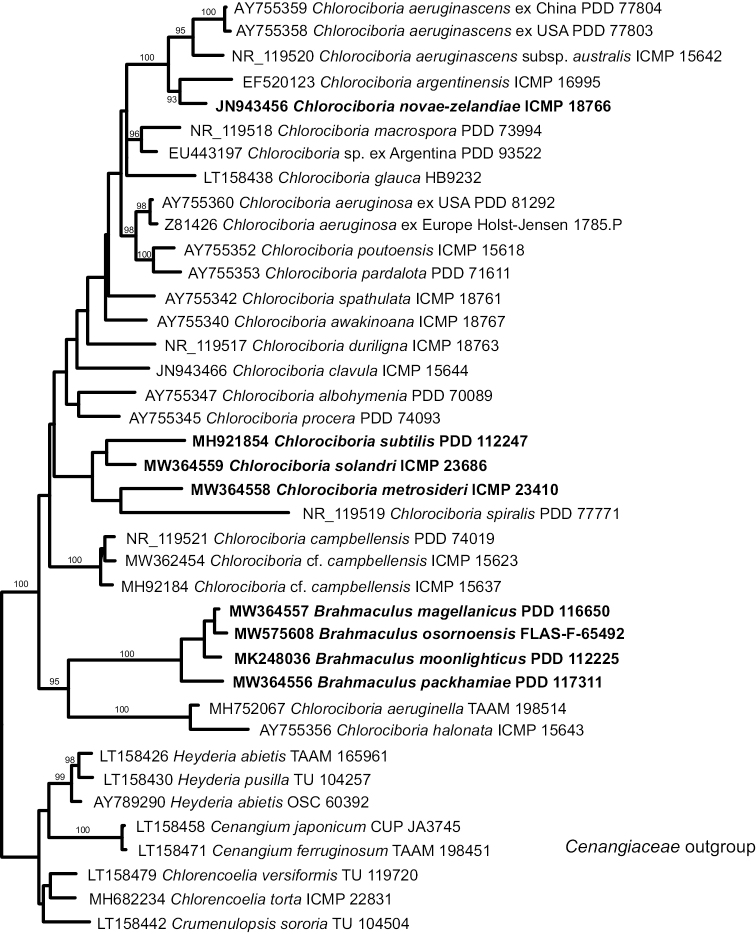
ML tree based on an ITS alignment, treating all *Chlorociboria* and *Brahmaculus* species with ITS sequences available. Taxa newly named in this paper in bold. Bootstrap values where >90%.

In both the multi-gene and ITS analyses, *Brahmaculus* forms a monophyletic clade within Chlorociboriaceae, but *Chlorociboria* as accepted here is paraphyletic (Figs 1, 2). The *Brahmaculus* species form a well-supported clade sister to a well-supported clade comprised of *Chlorociboria aeruginella* and *C. halonata* (from Northern Europe and New Zealand, respectively). The clade comprised of *Brahmaculus* plus these two species of *Chlorociboria* is sister to a clade containing the bulk of sequenced species of *Chlorociboria*, including the type species of the genus, *C. aeruginosa*.

At the species level, the ITS analysis supports the molecular phylogenetic distinctiveness of the novel species of *Brahmaculus* and *Chlorociboria* accepted here (Fig. 2).

### Taxonomy

#### Brahmaculus

Taxon classificationFungiHelotialesChlorociboriaceae

P.R.Johnst. gen. nov.

709B525D-C684-5438-92CC-E968997BFB11

838724

##### Type species.

*Brahmaculus moonlighticus* P.R.Johnst.

##### Etymology.

From Hindu mythology, named after Brahma, the four-headed creator god, reflecting the multiple heads of the apothecia, and the masculine diminutive -culus.

##### Diagnosis.

Phylogenetically Chlorociboriaceae, distinguished from *Chlorociboria* by its terrestrial habitat, and apothecium with stipe branched near apex, each branch with an apothecial cup.

##### Description.

Apothecia stipitate, yellow rhizomorphs at base of stipe, the stipe branched apically several times, each branch holding an apothecial cup. Receptacle and stipe densely covered with short hairs. Hairs more or less straight, cylindric, thin walled, with a few septa, pale brown intracellular pigment, externally densely encrusted with yellowish material, encrusting material dissolving in KOH + Melzer’s reagent. The hymenium within each apothecial cup is typically divided into smaller segments, with areas comprising asci and paraphyses separated by clumps of hair-like elements. Excipulum comprises cylindric cells arranged more or less parallel to the surface, cells mostly long-cylindric, but sometimes with outermost 1–2 layers of cells short and broad-cylindric, cell walls slightly thickened, hyaline, cells near base of hairs with pale brown vacuolar pigment. Asci with wall thickened at apex, amyloid pore extending through the wall, flaring near the inside and especially toward outside of the wall, 8-spored, with croziers. Paraphyses simple or tapering to apex, of similar length as asci. Ascospores oblong-elliptic, 0-septate, hyaline.

##### Notes.

The four species described below are phylogenetically distinct but remarkably similar morphologically. There appear to be small differences in size and colour of the apothecia and shape of the paraphyses and hairs but having only a single specimen available for each species makes the significance of these differences difficult to assess. The rhizomorphs at the base of the stipe appear to be associated with tree roots. Based on the collecting sites, in South America and New Zealand the roots are likely to be Nothofagaceae, in Australia they may also be Nothofagaceae but *Eucalyptus* species were also growing in the vicinity. Observations from the South American specimens showed a loose weft of mycelium around the Nothofagaceae roots but there was no clear evidence of a mantle or ectomycorrhizal association. It is possible that these fungi are root endophytes, or perhaps parasites of Nothofagaceae-associated ectomycorrhizal fungi.

#### Brahmaculus magellanicus

Taxon classificationFungiHelotialesChlorociboriaceae

M.E.Sm. & P.R.Johnst. sp. nov.

BE6E837C-6BF6-5A8A-A34A-687B505C60C3

838730

Figure 3

##### Typification.

Chile – **Magallanes** • Puente San Pedro, south of Punta Arenas, stream near the end of the road, *Nothofagus betuloides* forest; -53.6993, 70.9695; Alija Mujic (MES2454) leg.; 5 Apr 2017; SGO – ***Holotype***; FLAS-F-65086 – ***isotype***; PDD 116650 – ***isotype***.

##### Etymology.

Refers to the Magellanic forests of the type locality.

##### Diagnosis.

Phylogenetically distinct from other known *Brahmaculus* spp., apothecia 3–8 × 1–2.5 mm, paraphyses undifferentiated to rounded apex, ascospores 5.5–9 × 1.5–2 µm (average 7.3 × 1.7 µm).

##### Description.

Apothecia 3–8 mm high, stipe 0.4–0.6 mm wide, cap 1–2.5 mm wide, the more or less globose cap comprising several closely packed apothecial cups, these arising from short, branches at the top of the stipe, hymenium pale yellow, hymenial areas broken into smaller segments by groups of bright yellow, hair-like elements amongst the fertile parts of the hymenium. Receptacle densely covered with stiff, bright yellow hairs, stipe with shorter hairs. Hairs 45–70 × 2.5–4.5 µm, straight, cylindric, tapering gradually in apical half toward small, rounded apex, thin-walled, sparsely septate, pale brown vacuolar pigment, densely encrusted with coarse, bright yellow crystals that dissolve in KOH + Melzers. Ectal excipulum comprising narrow-cylindric cells 8–20 × 2.5–3 µm oriented at low angle to receptacle surface, wall slightly thickened, mostly hyaline except cells at the base of hairs have pale brown vacuolar pigment. Medullary excipulum similar in structure but cells wider, 4.5–8 µm diam. Paraphyses 1.5–2.5 µm diam., undifferentiated at rounded apex, about same length as asci. Asci 40–55 × 4–5 µm, cylindric, apex rounded, wall thickened, amyloid pore extends through the wall, diffuse and flaring slightly towards the outside of the wall, crozier at base, 8–spored. Ascospores 5.5–9 × 1.5–2 µm (average 7.3 × 1.7 µm, n = 12), oblong elliptic, ends rounded, flattened on one side, straight to slightly curved, 0-septate, hyaline.

**Figure 3.**
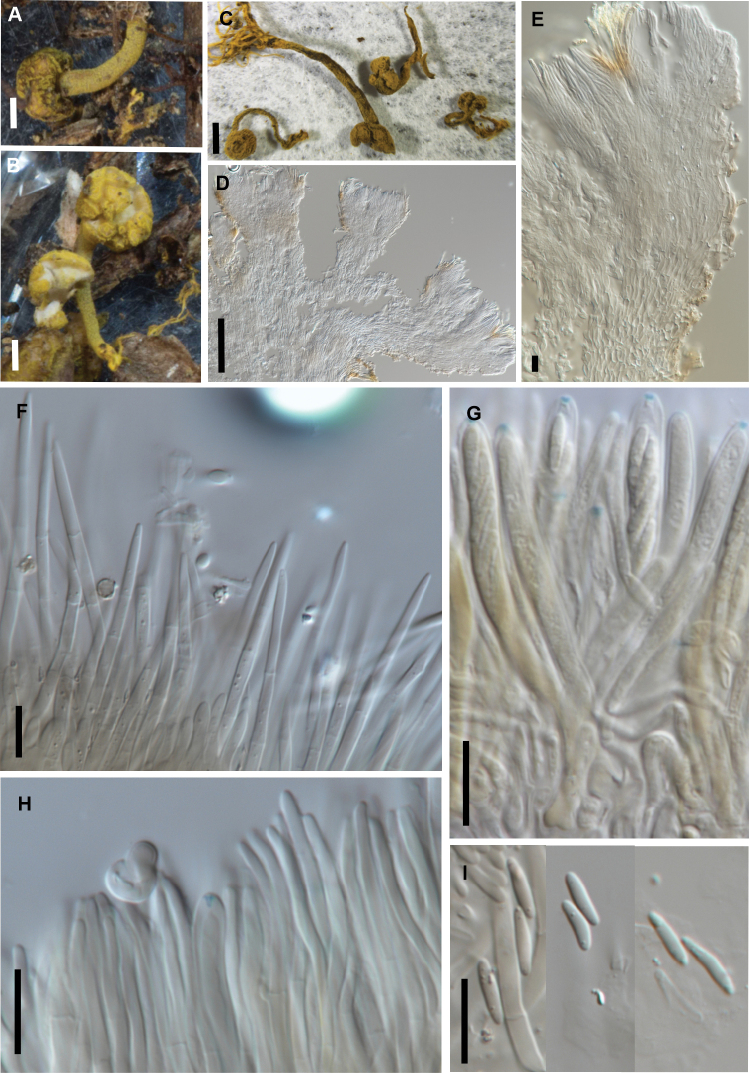
*Brahmaculus magellanicus* (PDD 116650) **A, B** fresh apothecia **C** dried apothecia **D** ascoma in vertical section, showing multiple apothecial cups on short branches **E** ascoma in vertical section showing excipular tissue and hairs, and a clump of hairs within the hymenium **F** squash mount showing hairs in KOH **G** asci **H** paraphyses **I** ascospores. Scale bars: 1 mm (**A–C**); 100 µm (**D**); 10 µm (**E–I**).

##### Notes.

The two Chilean species differ macroscopically, *B. magellanicus* having noticeably thinner stipes than *B. osornoensis*. The only known collection of *B. magellanicus* is from Magellanic subpolar forest in Patagonia that is dominated by *Nothofagus betuloides*. It is possible that this *Brahmaculus* species is restricted to these sub Antarctic cold southern forests but more specimens are needed to determine the range of the species.

#### Brahmaculus moonlighticus

Taxon classificationFungiHelotialesChlorociboriaceae

P.R.Johnst. sp. nov.

8114EF17-DDF2-5151-8A57-EA23E5E022C6

838733

Figure 4

##### Typification.

New Zealand – **Buller** • Moonlight Creek; -42.2713, 171.4587; on soil under Nothofagaceae; A. Chinn leg.; 10 May 2018; PDD 112225 – ***holotype***.

##### Etymology.

From the type locality. Historically important as a gold mining area (where T.H. Chinn, the great-great grandfather of the collector of the type specimen, prospected for gold in the 1880’s), the name also reflects the deep golden colour of the apothecia of this fungus.

##### Diagnosis.

Phylogenetically distinct from other known *Brahmaculus* spp., apothecia 1.5–3 × 1–1.8 mm, paraphyses taper slightly to rounded apex, ascospores 6.5–8.5 × 1.5–2(–3) µm (average 7.7 × 1.9 µm).

##### Description.

Apothecia 1.5–3 mm high, stipe 0.25–0.5 mm wide, cap 1–1.8 mm wide, bright golden-yellow when fresh, consistently with four short branches arising from top of stipe, each branch with its own apothecial cup, hymenium pale yellow, divided into a complex pattern with hymenial areas separated by narrow groups of golden yellow hair-like elements. Receptacle densely covered with stiff, bright yellow hairs. Hairs 40–60 × 3–4 µm, straight, cylindric, tapering slightly towards rounded apex, pale brown vacuolar pigment, wall smooth, encrusted with coarse yellow-brown crystals that dissolve in KOH + Melzer’s, few-septate. Ectal excipulum comprising long-cylindric cells 15–25 × 3–5 µm, but with the outermost 1–2 layers of cells short and broad-cylindric, 6–8 µm diam., cell walls slightly thickened, hyaline, cells near base of hairs with pale brown vacuolar pigment. Medullary excipulum comprising partly tangled hyphae 3–4 µm diam. with walls thin, hyaline. Paraphyses 2–3 µm diam., tapering slightly towards rounded apex, about same length as asci. Asci 45–55 × 5.5–6 µm, cylindric, tapering slightly to broad subtruncate apex, wall uniformly thickened across apex, amyloid pore extending through wall, flaring slightly towards both inside and outside of wall. Ascospores 6.5–8.5 × 1.5–2 (–3) µm (average 7.7 × 1.9 µm, n = 50), oblong-elliptic to subfusoid, sometimes tapering suddenly to a narrower lower half, ends rounded, flat one side in side view, sometimes slightly curved or sigmoid, 0-septate, hyaline.

**Figure 4.**
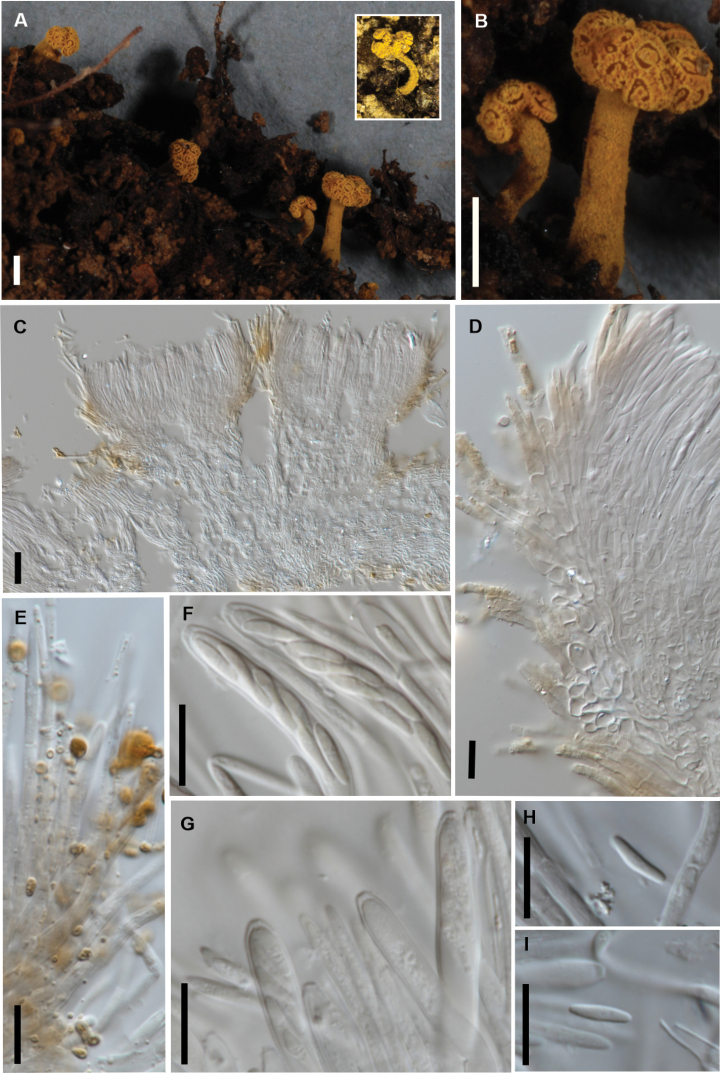
*Brahmaculus moonlighticus* (PDD 112225) **A** fresh apothecia (dried apothecium inset) **B** detail, fresh apothecia **C** ascoma in vertical section, showing multiple apothecial cups **D** ascoma in vertical section showing excipular tissue and hairs **E** hairs in squash mount in KOH **F** asci and ascospores **G** asci and paraphyses **H, I** ascospores. Scale bars: 1 mm (**A, B**); 100 µm (**C**); 10 µm (**D–I**).

##### Notes.

*Brahmaculus moonlighticus* has a stipe that consistently has 4 distinct branches near the apex. The other species have several separate hymenial cups, but these are held on very short branches arising from across the apex of the stipe, the margins of these cups superficially forming a more or less continuous layer.

#### Brahmaculus osornoensis

Taxon classificationFungiHelotialesChlorociboriaceae

M.E.Sm. & P.R.Johnst. sp. nov.

F89ADB04-3345-5A34-9C39-48FE3333B225

838734

Figure 5

##### Typification.

Chile • Parque Nacional Vicente Perez Rosales, Volcan Osorno, on the road to the ski area just above Mirador el Bosque, *Nothofagus dombeyi* forest; -41.1382, 72.5370; Matthew Smith and Alija Mujic (MES2942) leg.; 17 April 2017; SGO – ***holotype***; FLAS-F-65492 – ***isotype***; PDD 116649 – ***isotype***.

##### Etymology.

Refers to the type locality, Volcan Osorno.

##### Diagnosis.

Phylogenetically distinct from other known *Brahmaculus* spp., apothecia 3–6 × 1–2.5 mm, paraphyses taper slightly to rounded apex, ascospores 6.5–10(–11) × 1.5–2 µm (average 8.3 × 2 µm).

##### Description.

Apothecia 3–6 mm high, stipe 0.5–1 mm wide, cap 1–2.5 mm wide, the more or less globose cap comprising several closely packed apothecial cups, these arising from short, branches at the top of the stipe, hymenium pale yellow, hymenial areas broken into smaller segments by groups of bright yellow, hair-like elements amongst the fertile parts of the hymenium. Receptacle densely covered with stiff, bright yellow hairs, stipe with shorter hairs. Hairs 50–85 × 2.5–4 µm, straight, with a broad basal cell then cylindric, apically tapering suddenly to the narrow-rounded apex, thin-walled, sparsely septate, densely encrusted with coarse, bright yellow crystals that dissolve in KOH + Melzers. In squash mount, excipular cells broad-cylindric, about 15–30 × 8–12 µm, wall slightly thickened, hyaline. Paraphyses 2–2.5 µm, tapering slightly to rounded apex, about same length as asci. Asci 40–50 × 4–4.5 µm, cylindric, apex rounded, wall thickened, amyloid pore extends through the wall, flaring slightly towards the outside. Ascospores 6.5–10(–11) × 1.5–2.5 µm (average 8.3 × 2.0 µm, n = 50), oblong elliptic, ends rounded, one side flat in side view, sometimes slightly curved, 0-septate, hyaline.

**Figure 5.**
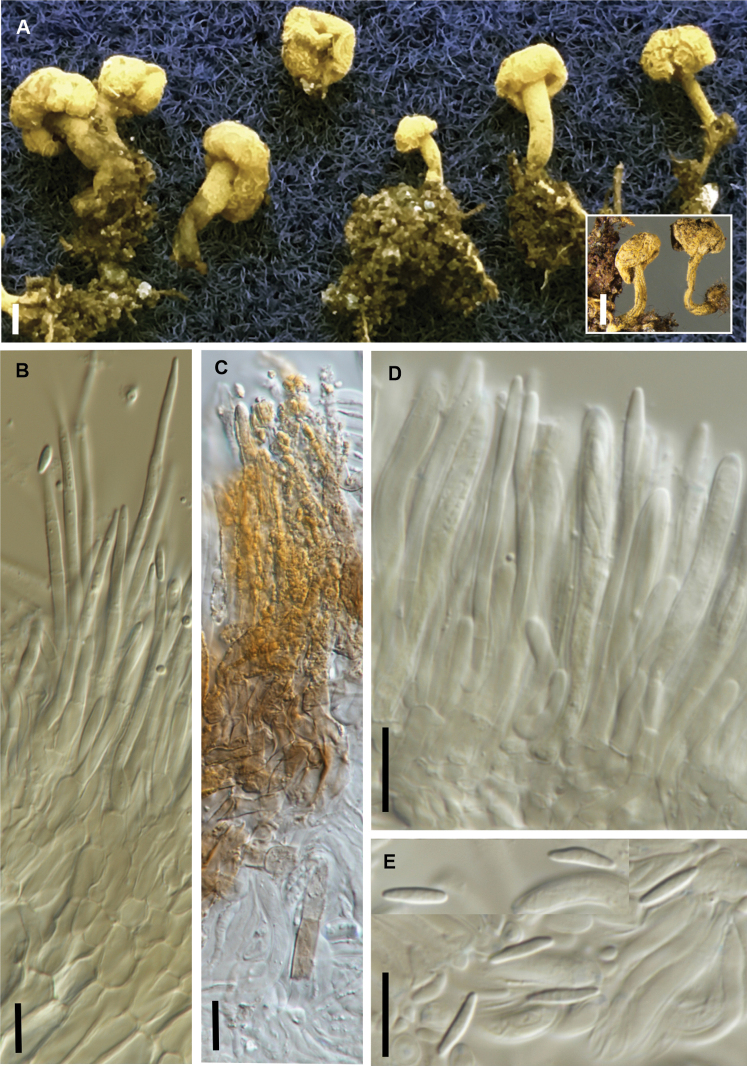
*Brahmaculus osornoensis* (PDD 116649) **A** fresh apothecia (dried apothecia inset) **B** squash mount, excipulum and hairs in 3% KOH + Melzer’s reagent **C** squash mount, hairs in water showing encrusting crystals **D** paraphyses and asci **E** ascospores. Scale bars: 1 mm (**A**); 10 µm (**B–E**).

##### Notes.

The two Chilean species differ macroscopically, *Brahmaculus osornoensis* having noticeably broader stipes than *B. magellanicus* and slightly longer ascospores. *B. osornoensis* is known only from *Nothofagus dombeyi* forest in northern Patagonia on Volcan Osorno in the Vicente Perez Rosales National Park. It is possible that this *Brahmaculus* species is restricted to the wetter and warmer forests in northern Patagonia, but more specimens are needed to determine the range of the species.

#### Brahmaculus packhamiae

Taxon classificationFungiHelotialesChlorociboriaceae

T.W.May & P.R.Johnst. sp. nov.

B1451E15-FEC1-5870-BF8E-AAF536CD4698

838729

Figure 6

##### Typification.

Australia – **Tasmania** • Geeveston District, Hermons Rd; -43.2652, 146.8613; J.M. Packham (6/R6/26) leg.; 5 June 1995; MEL 2363173 – ***holotype***; PDD 117311 – ***isotype***.

##### Etymology.

Named after the late Jillian (“Jill”) Mary Packham whose assiduous collecting activities detected the type collection.

##### Diagnosis.

Phylogenetically distinct from other known *Brahmaculus* spp., apothecia up to 11 × 2.5 mm, paraphyses undifferentiated to rounded apex, ascospores 5.5–8.5 × 1.5–2.5 µm (average 7.2 × 1.8 µm).

##### Description.

Apothecia up to 11 mm high, stipe up to 0.8 mm wide, cap up to 2.5 mm wide, the cap comprising several closely packed apothecial cups, these arising from short branches at the top of the stipe, hymenium white when fresh. Receptacle densely covered with stiff, bright yellow hairs, stipe with shorter hairs, yellow rhizomorphs at base. Hairs 40–60 × 2.5–3.5 µm, straight, narrow flask-shaped, broad near base then tapering suddenly to narrow-cylindric apical part, apex rounded, thin-walled, 1–2 septate near the base, densely encrusted with coarse, bright yellow crystals, that dissolve in KOH + Melzers. Ectal excipulum comprising cylindric cells 8–15 × 3–5 µm, oriented at a low angle to the receptacle surface, walls slightly thickened, hyaline. Medullary excipulum comprises partly tangled hyphae 3–5 µm diam. with walls thin, hyaline. Paraphyses 2–2.5 µm diam., undifferentiated at the rounded apex, about the same length as the asci. Asci 35–45 × 4.5–5.5 µm, cylindric, tapering slightly to broad, subtruncate apex, wall thickened across apex, amyloid pore extending through wall, flaring toward outside of wall. Ascospores 5.5–8.5 × 1.5–2.5 µm (average 7.2 × 1.8 µm, n = 20), oblong-elliptic, tapering slightly to rounded ends, one side flat in side view, sometimes slightly curved, 0-septate, hyaline.

**Figure 6.**
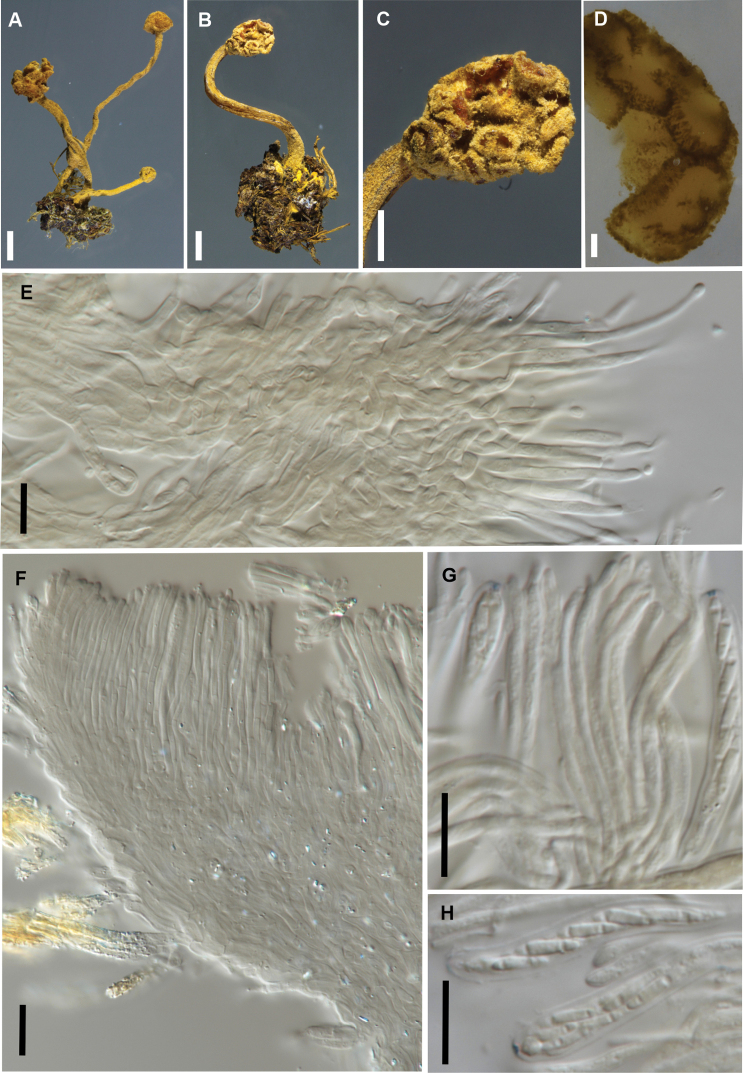
*Brahmaculus packhamiae* (PDD 117311) **A, B** dried apothecia **C** detail of head of dried apothecium **D** hymenial surface of rehydrated apothecium, showing multiple separate apothecial cups **E** squash mount showing excipular cells and hairs in KOH + Melzer’s reagent **F** ascoma in vertical section **G** paraphyses, asci, and ascospores **H** ascospores. Scale bars: 1 mm (**A–C**); 0.1 mm (**D**); 10 µm (**E–H**).

##### Notes.

*Brahmaculus packhamiae* is macroscopically and microscopically similar to the Chilean *B. magellanicus*, both species having relatively long and narrow stipes. Notes with the specimen, indicate that when fresh the ascomata “seem to be attached to roots”.

#### Chlorociboria metrosideri

Taxon classificationFungiHelotialesChlorociboriaceae

P.R.Johnst. sp. nov.

02DE039B-39AB-5DCD-A28B-1DCA5A2C26DD

838735

Figure 7

##### Typification.

New Zealand – **Bay of Plenty** • vic. Rotorua, Tarawera Falls (-38.1573, 176.5193); on fallen leaves *Metrosideros excelsa*; P.R. Johnston (D2565) leg.; 16 May 2019; PDD 116740 – ***holotype***; ICMP 23410 – ex type culture.

##### Etymology.

Refers to the host substrate of the known specimens.

##### Diagnosis.

Phylogenetically a *Chlorociboria*, differs in developing on dead leaves rather than wood and in the asci being 4-spored when mature.

##### Description.

Apothecia developing on partly decomposed fallen leaves, not associated with pigmentation of substrate. Apothecia less than 1 mm diam., sessile, with short, matted hairs around the margin, hymenium yellow. Hairs 20–45 × 4 µm, cylindric, walls thin, roughened. Apothecium in vertical section with ectal excipulum 30–40 µm wide, comprising short, broad-cylindric cells 5–7.5 µm diam., with walls hyaline, slightly thickened, rows of cells arranged at a high angle to the receptacle surface near the base of the cup, more parallel to the surface near edge of cup; medullary excipulum of narrow-cylindric cells with thin walls. Paraphyses 2–3 µm diam., taper slightly and gradually to rounded apex, extending 5–10 µm beyond asci. Asci 40–55 × 5.5–7 µm, cylindric, tapering slightly to broad, subtruncate apex, wall thickened at apex with amyloid pore extending as two narrow, parallel bands extending through the wall, initially with 8 spores, 4 spores aborting and 4–spored at maturity, crozier present. Ascospores 7.5–9.5 × 2.5–3.5 µm (average 8.3 × 3.1 µm, n = 20), oblong-elliptic, tapering to rounded ends, one side flat in side view, widest point towards one end, 0–septate, hyaline.

**Figure 7.**
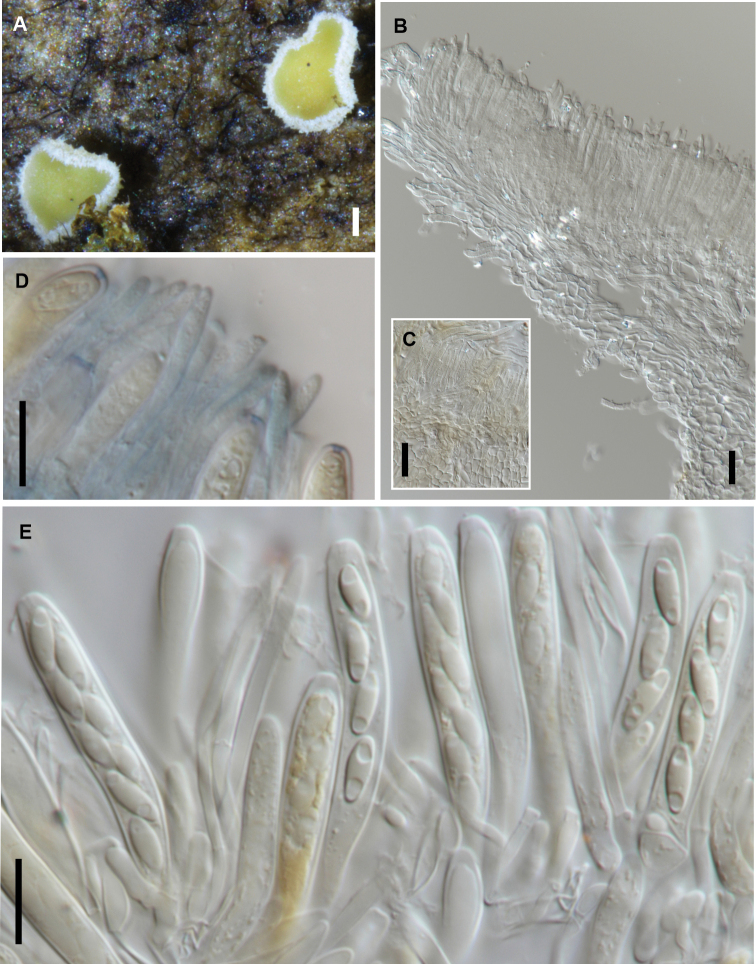
*Chlorociboria metrosideri* (PDD 116740) **A** fresh apothecia **B** margin of receptacle in vertical section **C** surface of receptacle in squash mount showing rough-walled hairs **D** apex of asci and paraphyses **E** immature ascus with 8 spores, and mature asci with 4 spores. Scale bars: 0.1 mm (**A**); 20 µm (**B, C**); 10 µm (**D, E**).

##### Additional specimen examined.

New Zealand – **Auckland** • Rangitoto Island, Kidney Fern Glen; -36.805544, 174.860064; on fallen, partly rotten *Metrosideros excelsa* leaves; P.R. Johnston (D2329) leg.; 23 Apr 2012; PDD 102723.

##### Notes.

The substrate in both specimens was partly rotted leaves. It is possible that this fungus has a broader host range as most host-specialised, leaf-inhabiting Leotiomycetes are found on recently fallen leaves of their preferred host. Cultures are slow growing (on PDA, 9 mm after 8 weeks) with sparse mycelium and pale brownish pigmentation, remaining sterile.

#### Chlorociboria novae-zelandiae

Taxon classificationFungiHelotialesChlorociboriaceae

P.R.Johnst. sp. nov.

4E6285D6-A886-55E5-A74E-8C817602034A

838736

##### Typification.

New Zealand – **Fiordland** • Kepler Track, control gates; -45.4396, 167.6822; on Nothofagaceae sp. dead wood; P.R. Johnston (D1484), R.E. Beever, S.R. Pennycook, R. Leschen, T. Lebel leg.; 10 May 2000; PDD 77447 – ***holotype***; ICMP 18766 – ex type culture.

##### Etymology.

Refers to the country of origin, in contrast to Argentina and the morphologically similar *C. argentinensis*, with which *C. novae-zelandiae* was previously confused.

##### Diagnosis.

Similar to *Chlorociboria argentinensis* in having small, allantoid ascospores and lacking tomentum hyphae, but phylogenetically distinct and with smaller ascospores and narrower asci.

##### Additional specimens examined.

***C. novae-zelandiae***: New Zealand – **Fiordland**. • Borland Lodge nature trail; on Nothofagaceae sp. dead wood; P.R. Johnston (D1471.2) leg.; 9 May 2000; PDD 77446 – **North Canterbury**. • Mt Thomas Forest, Richardson Track; on Nothofagaceae sp. dead wood; P.R. Johnston (D679) leg.; 15 Mar 1991; PDD 58574, ICMP 15616 – **Nelson.** • Arthur Range, Graham Valley Rd, track from Flora car park to Mt Arthur Hut; P.R. Johnston (D993) leg.; 6 May 1994; PDD 77444.

***C. argentinensis***: Argentina – **Tierra del Fuego** • Lago Fagnano, vic. Kosobo, road to hot springs; on *Nothofagus pumilio* fallen wood; P.R. Johnston (SA86), L. Lorenzo leg.; 22 Mar 1996; PDD 92026; ICMP 16994 – **Patagonia**. • Rio Negro, Nahuel Huapai National Park, path from Puerto Blest to Los Cantaros; on *Nothofagus dombeyi* fallen wood; P.R. Johnston (SA 188), I. Gamundí, C. Brion leg.; 2 Apr 1996; PDD 92027; ICMP 16995.

##### Notes.

Johnston and Park (2005: 690–693) provided a description of *C. novae-zelandiae*, from New Zealand specimens reported under the name *C. argentinensis*. Subsequent DNA sequencing of specimens from Argentina identified as *C. argentinensis*, showed that the New Zealand species is phylogenetically distinct. Morphologically, the two species are similar, both with an excipulum comprising highly gelatinous *textura intricata*, the apothecia lacking hair-like tomentum hyphae, and with small, allantoid ascospores. The New Zealand species has somewhat smaller ascospores (average 7.0 × 1.5 µm versus 9.9 × 1.9 µm) and narrower asci (3.5–4.5 µm versus 4–5.5 µm) compared with the Argentinian specimens recognised here as *C. argentinensis*. The Argentinian specimens match closely the description of Dixon (1975).

#### Chlorociboria solandri

Taxon classificationFungiHelotialesChlorociboriaceae

P.R.Johnst. sp. nov.

F19D43CA-04BF-5257-A33A-E12C54E718D9

838737

Figure 8

##### Typification.

New Zealand – **Fiordland** • Fiordland National park, Kepler Track, Rainbow Reach; -45.4429, 167.6802; on *Fuscopora solandri* fallen leaves; P.R. Johnston (D686) leg.; 17 Mar 1991; PDD 58580 – ***holotype***; ICMP 23686 – ex-type culture.

##### Etymology.

Refers to the host substrate of the holotype.

##### Diagnosis.

Phylogenetically a *Chlorociboria*, developing on fallen leaves rather than wood, differs from *Chlorociboria metrosideri* in having flexuous, coiled hairs and lanceolate paraphyses.

##### Description.

Apothecia developing on fallen leaves, not associated with any pigmentation of substrate. Apothecia less than 1 mm diam., short-stipitate, receptacle densely covered with short, white hairs, hymenium pale yellow. Hairs 30–40 × 3–4 µm, short-cylindric, undifferentiated to apex, septate, thin-walled, roughened all over, flexuous, coiled and tangled. Apothecia in vertical section with ectal excipulum 45 µm wide, comprising short-cylindric to subglobose cells 5–8 µm diam. oriented at high angle to receptacle surface, with walls hyaline, thickened, agglutinated, amyloid in some specimens. Medullary excipulum non-gelatinous, comprising narrow-cylindric hyphae with thin walls. Tissue at base of stipe of gelatinous *textura intricata*. Paraphyses up to 5 µm diam., lanceolate, tapering to narrow rounded apex, extending 20–30 µm beyond asci, wall distinctively thickened at base, amyloid in some specimens. Asci 40–55 × 4.5–5.5 µm, cylindric, tapering gradually to small, subtruncate apex, wall thickened at apex, amyloid pore extending through wall, flaring towards outside of wall, crozier present, 8–spored. Ascospores 8–11.5 × 1.5–2 µm (average 10.0 × 1.7 µm, n = 20), oblong-elliptic to subfusoid, widest point slightly towards the upper end, taper to narrow-rounded ends, 0–septate, hyaline.

**Figure 8.**
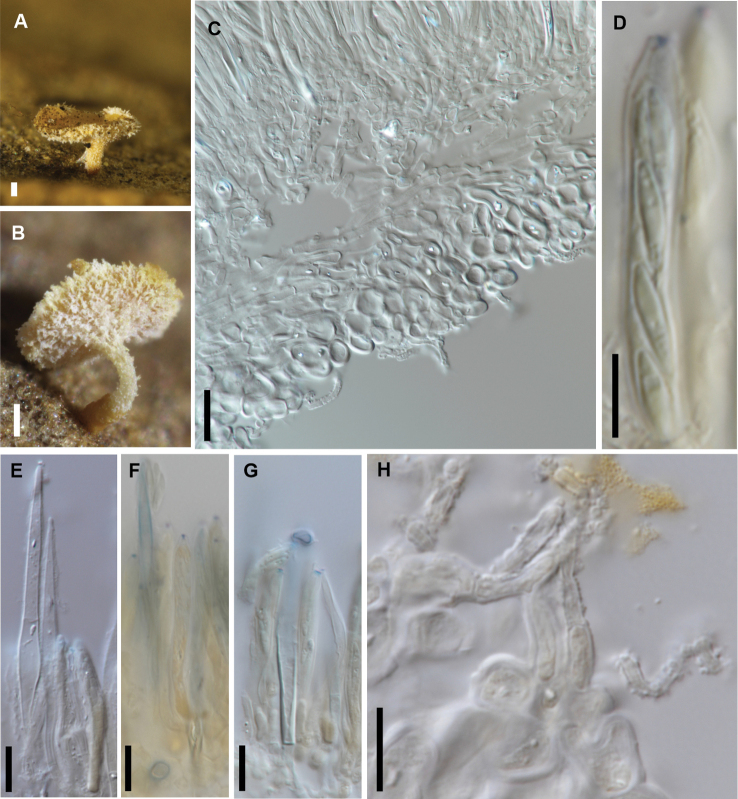
*Chlorociboria solandri***A** dried apothecium **B** fresh apothecium **C** margin of receptacle in vertical section **D** ascospores **E** paraphyses **F** amyloid paraphysis **G** base of paraphysis with thick wall **H** details of coiling, rough-walled excipular hairs. Images: PDD 58580 (**A, C, D, F–H**); PDD 61833 (**B, E**). Scale bars: 0.1 mm (**A, B**); 20 µm (**C**); 10 µm (**D–H**).

##### Additional specimens examined.

New Zealand – **Taupo** • Kaimanawa Forest Park, Tree Trunk Gorge; on *Fuscopora solandri* fallen leaves; P.R. Johnston (D877), I. Gamundí leg.; 1 Feb 1993; PDD 61833 – **Mid Canterbury** • Craigieburn, Cave Stream; on *Fuscopora solandi* fallen leaves; E. Horak leg.; 31 Mar 1983; PDD 92925.

##### Notes.

*Chlorociboria solandri* is micromorphologically distinctive in having scattered, large, lanceolate paraphyses, short-cylindric to more or less globose, thick-walled excipular cells, excipular tissue reacting either blue or red to Melzer’s reagent, and coiling, rough-walled hairs. Known from two specimens from *Fuscopora solandri* leaves. A third specimen in poor condition, PDD 92925, could be the same species; it is morphologically similar but has longer hairs than the other two specimens. Cultures on agar are very slow growing (10 cm after 4 weeks), have little aerial mycelium and pale yellow brown pigments, remaining sterile.

#### Chlorociboria subtilis

Taxon classificationFungiHelotialesChlorociboriaceae

P.R.Johnst. sp. nov.

1A8DC5DF-63BC-5459-BA4B-2C0562A490F0

838738

Figure 9

##### Typification.

New Zealand – **Westland** • Haast Pass Summit, Lookout Track; -44.1063, 169.3519; on fallen leaves *Dracophyllum* sp.; P.R. Johnston (D2515), M. Padamsee leg.; 16 May 2018; PDD 112247 – ***holotype***.

##### Etymology.

From subtilis (delicate) referring to the stature of the apothecia.

##### Diagnosis.

Blue-green apothecia on blue-green stained fallen, partly decomposed leaves, hairs on receptacle rough-walled, somewhat flexuous, ascospores filiform, 45–55 × 1 µm.

##### Description.

Apothecia erumpent from blue-green stained leaf tissue. Apothecia less than 1 mm diam., cupulate with short, broad stipe, receptacle pale blue-green with tangled, white hairs, especially near the edge of the cup. Hairs 55–75 × 3–4 µm, somewhat flexuous, wall roughened. Apothecia in vertical section with ectal excipulum up to 30 µm wide, cells 6–10 µm diam., short-cylindric to square, walls thick, cells arranged in rows with a high angle to the receptacle surface; medullary excipulum poorly developed, two or three rows of narrow-cylindric cells, walls encrusted with blue-green material; stipe with thick-walled *textura intricata*. Paraphyses 1.5–2 µm diam., slightly wider towards the apex, often branched in the upper 20–30 µm, extending 15 µm beyond asci. Asci 85–105 µm × 5.5–6.5 µm cylindric, tapering gradually to small, truncate apex, wall thickened at apex, amyloid pore in inner half of wall, reaction most intense on inner edge of wall, pore appears more or less U-shaped, sloping outwards slightly through the wall, 8-spored, spores confined to the upper 60–100 µm of ascus, crozier present. Ascospores 45–55 × 1 µm, filiform, straight, 0–septate, hyaline.

**Figure 9.**
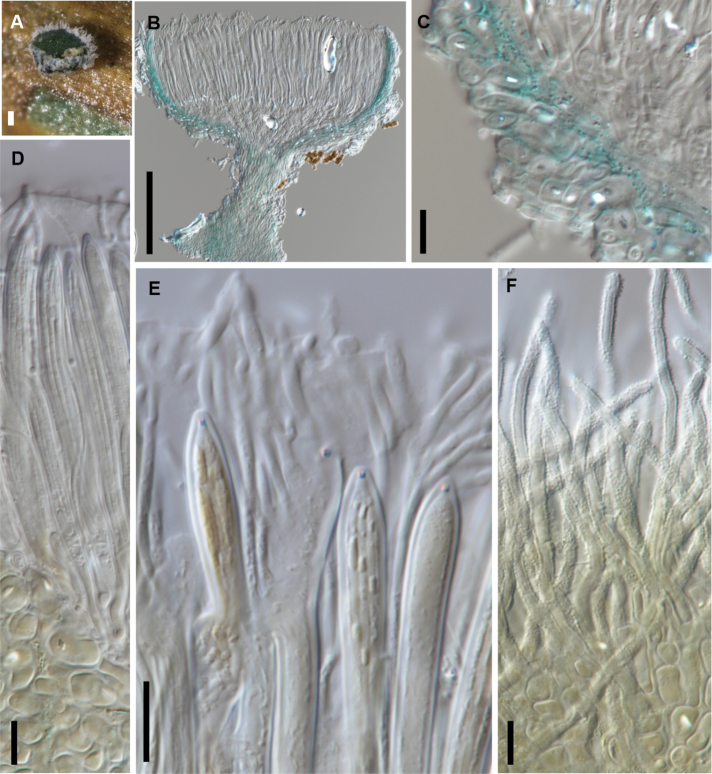
*Chlorociboria subtilus* (PDD 112247) **A** dried apothecium **B** apothecium in vertical section **C** detail of margin of receptacle in vertical section **D** asci, ascospores and paraphyses **E** detail of apex of paraphyses and asci **F** excipular hairs (squash mount). Scale bars: 0.1 mm (**A**); 100 µm (**B**); 10 µm (**C–F**).

##### Additional specimens examined.

New Zealand – **Nelson** • Arthur Range, Graham River Valley Rd, track from Flora car park to Mt Arthur Hut; on *Dracophyllum pyrimidale* fallen leaves; P.R. Johnston (D990) leg.; 6 May 1994; PDD 105292 – **Central Otago** •vic. Dunedin, Great Moss Swamp; on *Dracophyllum uniflorum* fallen leaves; P.R. Johnston (D82) leg.; 12 May 1984; PDD 105293 – **Mid Canterbury** • Craigieburn, Cave Stream; on *Dracophyllum uniflorum* fallen leaves; P.R. Johnston (D248) leg.; 23 Feb 1988; PDD 105294 – **Taupo** • Tongariro National Park, Ohakune Mountain Road, Blyth Track; on *Fuscopora cliffortioides* fallen leaves; P.R. Johnston (D353) leg.; 20 May 1989; PDD 55523 • Rangitoto Station, Ranginui Summit; on *Dracophyllum pyrimidale* fallen leaves; P.R. Johnston (D1622), S.R. Whitton leg.; 6 May 2001; PDD 117584.

##### Notes.

Most specimens are on fallen leaves of *Dracophyllum* spp., but the host range may be more extensive. A specimen on *Fuscopora cliffortioides* (PDD 55523) is morphologically similar, but perhaps with longer ascospores.

## Discussion

The *Brahmaculus* species described here are so morphologically and ecologically divergent from *Chlorociboria* that they must be placed in their own genus. All four new species are members of a well-supported monophyletic lineage within Chlorociboriaceae (Fig. 2). However, in both the multigene and ITS analyses (Figs 1, 2) the *Brahmaculus* clade makes *Chlorociboria*, as currently understood in a morphological sense, paraphyletic. If alternative generic limits were to be drawn to recognise only monophyletic genera within Chlorociboriaceae, it is unclear how these genera could be distinguished morphologically. The type of *Chlorociboria* (*C. aeruginosa*) sits within the main *Chlorociboria* clade, and hence the name *Chlorociboria* will remain attached to the bulk of the species so far described in the genus. However, further sampling of *Chlorociboria*, including of species lacking green pigments (see below) is required before redrawing generic limits, especially in regard to the distinguishing morphological characters of the main *Chlorociboria* clade in relation to the phylogenetically differentiated species *C. halonata* and *C. aeruginella*.

The multi-gene phylogeny places Chlorociboriaceae in an isolated position near the base of Helotiales. Earlier analyses had suggested a relationship between Chlorociboriaceae and Cyttariaceae (Peterson and Pfister (2010a). The multiple genes newly available from a *Cyttaria nigra* specimen (PDD 117571) allowed Cyttariaceae to be treated in the multi-gene analysis. This showed that although Cyttariaceae was similar to Chlorociboriaceae in having an isolated position near the base of Helotiales, no particular phylogenetic relationship was found between the families. Cyttariales is treated here as a synonym of Helotiales.

Direct observations of the mycelium at the base of the stipes of several of the *Brahmaculus* spp. suggests a biotrophic relationship with either the roots of Nothofagaceae (possibly as root endophytes), or the mycorrhizal fungi associated with those roots (possibly as parasites). Johnston and Park (2005) noted a possible ecological relationship between wood rotting basidiomycetes and some of the wood-inhabiting *Chlorociboria* spp.

Not all of the specimens accepted here as *Chlorociboria* develop green pigment on their substrate. These include *C. glauca* and two of the newly described species from New Zealand (*C. metrosideri* and *C. solandri*). Both of these newly described species develop on fallen leaves, they have whitish rather than green apothecia, form no green pigment on their substrate, but have an excipular structure and the short, rough-walled, hair-like elements typical of several of the New Zealand representatives of the genus. The third newly named species from New Zealand, *Chlorociboria subtilis*, also develops on fallen leaves, but both the apothecia and the adjacent parts of the leaf have a blue-green pigment. The apothecial hairs of this species are better developed than those of most *Chlorociboria* species. Fungi morphologically similar to *C. subtilis* occur on fallen leaves in both eastern Australia (e.g. PDD 117581) and southern South America (unpubl. data) but they are not named here as only small specimens, and no DNA sequences, are available for these fungi.

Most known *Chlorociboria* species develop on green-stained, fallen wood. It is likely that there are other unrecognised *Chlorociboria* species, placed in other genera because they lack green pigment and have substrates apart from wood, the visually obvious features historically regarded as characteristic of *Chlorociboria*. Their true phylogenetic relationship may be revealed only when DNA sequence data becomes available for them, unless an alternative set of morphological features is discovered that is found to be characteristic of the Chlorociboriaceae clade. Huhtinen et al. (2010) discuss other seemingly ecologically or morphologically atypical *Chlorociboria* spp. from Europe. If these are shown to be *Chlorociboria* phylogenetically, they may be key to discovering phylogenetically informative morphological characters for the genus and family.

## Conclusions

The phylogenetic breadth of Chlorociboriaceae is becoming better understood with the identification of *Brahmaculus* as a distinct lineage. For *Chlorociboria*, recognising that not all species form apothecia on green-stained wood is an important step in characterising the genus and family both morphologically and phylogenetically, and in resolving more accurately its geographic distribution globally.

## Supplementary Material

XML Treatment for Brahmaculus

XML Treatment for Brahmaculus magellanicus

XML Treatment for Brahmaculus moonlighticus

XML Treatment for Brahmaculus osornoensis

XML Treatment for Brahmaculus packhamiae

XML Treatment for Chlorociboria metrosideri

XML Treatment for Chlorociboria novae-zelandiae

XML Treatment for Chlorociboria solandri

XML Treatment for Chlorociboria subtilis
